# Low-Intensity Exercise Routine for a Long Period of Time Prevents Osteosarcopenic Obesity in Sedentary Old Female Rats, by Decreasing Inflammation and Oxidative Stress and Increasing GDF-11

**DOI:** 10.1155/2021/5526665

**Published:** 2021-07-19

**Authors:** Beatriz Mena-Montes, David Hernández-Álvarez, Gibrán Pedraza-Vázquez, Rafael Toledo-Pérez, Raúl Librado-Osorio, Jorge Antonio García-Álvarez, Adriana Alarcón-Aguilar, Roberto Lazzarini-Lechuga, Oscar Rosas-Carrasco, Mina Königsberg, Norma Edith López-Diazguerrero, Armando Luna-López

**Affiliations:** ^1^Posgrado en Biología Experimental, DCBS, Universidad Autónoma Metropolitana Iztapalapa, Ciudad de México, Mexico; ^2^Laboratorio de Bioenergética y Envejecimiento Celular, Departamento de Ciencias de la Salud, Universidad Autónoma Metropolitana Iztapalapa, Ciudad de México, Mexico; ^3^Deparmento Investigación Básica, Instituto Nacional de Geriatría, Ciudad de México, Mexico; ^4^Doctorado en Ciencias Biológicas y de la Salud, DCBS, Universidad Autónoma Metropolitana, Ciudad de México, Mexico; ^5^Facultad de Ciencias, Universidad Nacional Autónoma de México, Ciudad de México, Mexico; ^6^Departamento de Biología de la Reproducción, Universidad Autónoma Metropolitana Iztapalapa, Ciudad de México, Mexico; ^7^Departamento de Investigación Clínica-Epidemiológica, Instituto Nacional de Geriatría, Ciudad de México, Mexico; ^8^Departamento de Ciencias de la Salud, Universidad Iberoamericana, Ciudad de México, Mexico

## Abstract

The loss of skeletal muscle mass and strength is known as sarcopenia; it is characterized as a progressive and generalized muscle disorder associated with aging. This deterioration can seriously compromise the elderly's health and reduce their quality of life. In addition to age, there are other factors that induce muscle mass loss, among which are sedentary lifestyle, chronic diseases, inflammation, and obesity. In recent years, a new clinical condition has been observed in older adults that affects their physical capacities and quality of life, which is known as osteosarcopenic obesity (OSO). Osteoporosis, sarcopenia, and obesity coexist in this condition. Physical exercise and nutritional management are the most widely used interventions for the treatment and prevention of sarcopenia. However, in older adults, physical exercise and protein intake do not have the same outcomes observed in younger people. Here, we used a low-intensity exercise routine for a long period of time (LIERLT) in order to delay the OSO appearance related to sedentarism and aging in female Wistar rats. The LIERLT routine consisted of walking at 15 m/min for 30 min, five days a week for 20 months. To evaluate the effects of the LIERLT routine, body composition was determined using DXA-scan, additionally, biochemical parameters, inflammatory profile, oxidative protein damage, redox state, and serum concentration of GDF-11 at different ages were evaluated (4, 8, 12, 18, 22, and 24 months). Our results show that the LIERLT routine delays OSO phenotype in old 24-month-old rats, in a mechanism involving the decrease in the inflammatory state and oxidative stress. GDF-11 was evaluated as a protein related to muscle repair and regeneration; interestingly, rats that perform the LIERLT increased their GDF-11 levels.

## 1. Introduction

Aging is a natural and irreversible process that is characterized by the progressive decline of individual's physiological, biochemical, and structural functions, which makes the organisms more susceptible to acquiring chronic degenerative diseases related to aging, such as diabetes, obesity, osteoporosis, neurodegenerative diseases, and sarcopenia [1]. Skeletal muscle is one of the main tissues that deteriorate during aging; this deterioration brings with it a decrease in muscle mass and strength, which is known as sarcopenia. Muscle mass and strength gradual loss are associated with a high probability of adverse health outcomes including falls, fractures, physical disability, and mortality [2]. In addition to age, there are other factors that might increase skeletal muscle mass loss, among which are a sedentary lifestyle, obesity, chronic diseases, and low-grade chronic inflammation [3, 4]. One of the main risk factors for developing sarcopenia is obesity. It is known that during aging the total body fat mass increases while the muscle mass and bone mineral density decrease. These changes are independent of physiological fluctuations in weight and body mass index (BMI) [4]. Different phenotypes related to the increase in body fat and its effects on other tissues, such as the muscle and bone have been described. A very well-studied phenotype is sarcopenic obesity (OS), where the negative effects of fat on muscle composition and functionality have been determined. This topic has been of considerable interest to researchers from different countries, due to its high association with the health conditions of older adults. Nevertheless, OS definition, evaluation, prevalence, and cut-off points are issues that are still being defined. In recent years, two systematic reviews on OS during aging have highlighted the differences in the evaluation methodologies, the ethnic groups, and the gender as important issues that diversify and complicate OS understanding in humans. Another problem is the prevalence, since large variations have been reported; however, it is known that during aging there is a higher OS occurrence in women, due to the accumulation of visceral fat during postmenopause [5–8]. Moreover, a decrease in bone mass density, muscle mass, and strength has been observed in postmenopausal women, explaining the higher incidence of OS compared to men [9]. Hence, since the highest incidence of this disease is in women, we decided to use female rats for our study, in contrast to most animal studies conducted in males.

Another interesting phenotype, which is characterized by the combination of sarcopenia, obesity, osteoporosis, and strength loss is known as osteosarcopenic obesity (OSO). OSO has a serious impact on the elderly life quality and predisposes this population to serious metabolic disorders such as insulin resistance, anabolic hormones reduction, and chronic inflammation [9–12]. OSO prevalence is expected to increase in the coming years, thus becoming a serious public health problem, mainly in societies that have highly hypercaloric diets and a high level of sedentary lifestyle.

Physical exercise and nutritional management, mainly protein and/or amino acid supplementation, are currently the most widely used interventions for the treatment and prevention of sarcopenia. The PROVIDE study showed that oral protein supplementation, enriched with vitamin D and Leucine, for 13 weeks was a successful intervention to improve muscle mass and strength in sarcopenic older adults [13–16]. Despite those benefic effects, it is known that physical exercise and protein intake do not have the same outcomes in the elderly than the ones observed in younger people, due to a phenomenon known as metabolic resistance. To counteract metabolic resistance, it is necessary to increase protein consumption in a range between 25 and 150% more than what a healthy adult consumes [17–19]. Besides, various studies that have been conducted to determine protein intake effectiveness in the elderly population are biased, since they were performed recruiting healthy older adults without functional limitations and not individuals with protein intakes lower than the recommended daily dose [20]. On the other hand, it has been reported that physical training on a regular basis promotes and maintains the health and life quality of the elderly. The exercise prescription in older adults depends on their health condition, and the beneficial effects will depend on the exercise type, as well as its duration and intensity [21, 22]. Performing an exercise routine has been shown to bring multiple benefits, particularly in frail obese older adults, as it delays the loss of muscle mass [23].

In recent years, the search for molecules that could delay the aging deleterious effects has augmented, in particular for those molecules that increase tissue repair and regeneration. An important molecule that has been studied for its rejuvenating function is the growth and development factor 11 (GDF-11). Historically, GDF-11 was described in studies using the heterochronic parabiosis model, where GDF-11 was shown to reverse aging-related cardiac hypertrophy [24]. Since the levels of this molecule were reported to decrease with age, Sinha and coworkers administered GDF-11 and managed to restore skeletal muscle structural and functional characteristics and increased its ability to perform strength and endurance exercises [25].

Furthermore, De Domenico et al. reported that GDF-11 levels increased after a 12-week exercise routine in 3-month-old mice, but not in 18-month-old mice [26]. Others have shown that a 6-week exercise routine augmented GDF-11 mRNA expression levels in aged mouse soleus muscle. Interestingly, in this work, the authors commented that the exercise-routine time was insufficient to observe changes in the muscle structure [27]. Thus, the aim of this work was to study the effects of a low-intensity exercise routine for a long period of time (LIERLT) in female rats of the Wistar strain, with OSO associated to sedentarism and aging. LIERLT performed for 20 months delayed OSO phenotype, decreased the inflammatory and oxidative stress states, and was related to increased expression levels of the regenerative protein GDF-11.

## 2. Materials and Methods

### 2.1. Animals

One hundred and five female Wistar rats (*Rattus norvegicus*) obtained from the animal colony of the Universidad Autónoma Metropolitana, Unidad Iztapalapa, were used. The animals were maintained with a 12/12-hour dark light cycle, with ad libitum food (Abene BDL-7100) and water. All animals' procedures were strictly carried out according to Mexican Official Ethics Standard NOM-062-ZOO-1999, and the Standard for the disposal of biological waste (NOM-087-ECOL-1995). The protocol was approved by the research ethics committee of the National Institute of Geriatrics and complies with NOM-062-ZOO-1999. At 4 months of age, the rats were divided into two groups, sedentary and exercised. Subsequently, determinations were made in the different stages of rats development and during aging as follows: young adults (4-8 months), adults (8-12 months), middle age (12-18 months), and old (18-24 months).

### 2.2. Sedentary Group (SED)

The sedentary group consisted of 60 rats. Each cage housed five rats. The rats were considered sedentary when they grew under standard conditions throughout their lives, with ad libitum food, and without any enrichment additions that could modify their mobility, as reported elsewere [28, 29]. The cage used for animal's accommodation was made of 6 mm thick transparent acrylic with the following measures: 43 × 53 × 20 cm. Rats lived there from 4 to 24 months old, when they were euthanized.

### 2.3. Low-Intensity Exercise Routine for a Long Period of Time (LIERLT)

At the age of 4 months, 45 female Wistar rats started with the recognition and adaptation to the training equipment (Treadmill Control LE8710 Panlab Harvard Apparatus) at a speed of 6 m/min for 10 min the first week, 9 m/min for 15 min the second week, 12 m/min for 20 min the third week, and 15 m/min for 30 min the four week. When the rats were adapted, the speed and time to perform the low-intensity physical routine was adjusted to a speed of 15 m/min, for 30 min, during 5 days of the week, until the rats reached the age of 24 months;at this age, the rats are considered old and were euthanized [30].

### 2.4. BMI

The body mass index (BMI) was determined by quantifying the body weight of each rat and was related to the nasorectal length (g/cm^2^). BMI determinations were made at 4, 8, 12, 18, 22, and 24 months of age. The growth curves were established using a polynomial adjustment. The growth curves of the female Wistar rats allow us to establish the different stages of development throughout life. 4-8 month old is considered a young adult; 8-12 month old is considered an adult; 12-18 month old is considered a middle age; 18-24 month old is considered old [28, 29]. Subsequently, a body composition analysis was performed using a DXA-scan.

### 2.5. Dual Energy X-Ray Absorptiometry (DXA-Scan)

The SED and LIERLT animal groups were analyzed by dual energy X-ray absorptiometry at the ages of 4, 8, 12, 18, 22, and 24 months (Discovery QDR Series, Hologic® Discovery 87899). The equipment was previously calibrated with the phantom (Hologic Rat Step Phantom P/N 010-0758) for all measurements. The animals were previously anesthetized with the ketamine (2.5 mL), xylazine (0.8 mL), and saline (2.5 mL) cocktail, in a dose of 100 *μ*L/100 g of weight. To avoid biases due to the size relationship between juvenile, adult, and aged individuals, body composition was reported as a percentage. Total fat mass was related to the total weight of the individual. This was also done with the bone mineral density (BMD). The percentage of fat and the lean mass without bone (LMWB) were determined by quantifying the fat free mass and subtracting and the bone content, to perform the relationship with respect to the total weight of the individual. The skeletal muscle index (SMI) was calculated as follows: the lean mass without bone of the four limbs was divided by the nasorectal length squared for each of the animals. The cut-off point to determine that *obesity* has been established in our OSO model was set when the percentage of body fat is greater than 30%. The cut-off point for *osteoporosis* was established when the BMD values were less than 2.5 times the SD with respect to the mean value of young adult rats (8 months) [31–35]. *Sarcopenia* and *dinapenia* were established when the percentage of LMWB and strength decreased below 2 times the SD with respect to the mean value of young adult rats.

### 2.6. Grip Strength

The grip strength was evaluated using a Rhino BAC-20 digital dynamometer (PKCh) with a measuring range between 0.1 N and 200 N (Newton) [36]. The dynamometer was vertically attached to a stainless steel grid and the maximum gripping force was recorded at the time when rat was released from the grid. The results are reported as N/kg-rat weight.

### 2.7. Biochemical Parameter Determination

After an 8-hour fast, the SED and LIERLT rats at the ages of 4, 8, 12, 18, 22, and 24 months were euthanized by decapitation. The gastrocnemius muscle was extracted and stored at -80°C. The blood was collected by a funnel. The serum was separated by centrifugation at 3500 rpm for 10 min. Glucose, creatinine, triglycerides, cholesterol, GOT, GPT, GGT, and HDL were determined in the serum using the SPOTCHEM EZ SP-4430 chemistry analyzer Arkray.

### 2.8. GDF-11 Serum Concentration

GDF-11 levels were determined in serum by ELISA using the commercial kit of the Cloud Clone Corp brand ELISA kit (# cat. SEC113Ra), following the manufacturer's recommendations.

### 2.9. Redox State GSH/GSSG Ratio

The concentration of reduced and oxidized glutathione (GSH and GSSG) was analyzed by high-performance liquid chromatography (HPLC) as previously described [36]. 100 mg of the gastrocnemius muscle was homogenized with 800 *μ*L hydrochloric acid/BPDS (10% HCL/1 mM BPDS). The suspension was centrifuged at 10,000 rpm for 5 min at 4°C, and the supernatant was recovered. 30 *μ*L of each sample were injected into the HPLC system. A 1525 Waters binary pump was used, coupled to a 2489 UV/vis detector calibrated at 210 nm; the stationary phase consisted of a Zorbax Eclipse XDB-C18 column of 4.6 × 250 mm and 5 *μ*M of particle and as a mobile phase 1% acetonitrile and 99% of a monobasic potassium phosphate buffer (20 mM of KH_2_PO_4_), pH 2.7, was used an isocratic flow of 1 mL/min was used. The samples were analyzed by ultraviolet detection at 210 nm. The area under the curve was determined using GSH and GSSG standards.

### 2.10. Protein Oxidative Damage

Proteins oxidation was determined by spectrophotometry in 96-well plates using the 2,4-dinitrophenylhydrazine reagent (DNPH). 20 *μ*L of DNPH (10 mM in 0.5MH_3_PO_4_) was mixed with 20 *μ*L of the sample and incubated in the dark for 10 min. Subsequently, 10 *μ*L of NaOH (6 M) was added and incubated for 10 min in the dark. Absorbance was measured at 450 nm. Carbonyl content was calculated as (Abs450/E)/total protein content of the sample [37].

### 2.11. Inflammatory Profile

Twenty-three cytokines IL-1*α*, IL-1*β*, IL-2, IL-4, IL-5, IL-6, IL-7, IL-10, IL-12p70, IL-13, IL-17A, IL-18, MCP-1, MIP-1a, MIP-3a, M-CSF, IFN-*γ*, G-CSF, GM-CSF, GRO-KC, RANTES, VEGF, and TNF-*α* were quantified from 50 *μ*L serum from sedentary or exercised rats using the commercial Bio-Plex Pro™ Human Cytokine 23-plex Assay (Bio-Rad Hercules CA) as described by the manufacturer. The quantification is based on the quantity of proteins adhered to their specific antibodies as previously reported [38]. To represent the values obtained in the heat map, the concentrations obtained from each of the cytokines were normalized with respect to the concentrations obtained from the young rats (4 months old). Subsequently, a logarithmic function was applied to the values obtained and plotted on a scale from -1 to 1. The TNF-*α*/IL-10 and IL-6/IL-10 ratios were calculated from the concentration of each cytokine.

### 2.12. Statistical Analysis

Data are presented as a mean ± standard error of each animal group (*n* = 5). The population growth curves were established by polynomial correlations, for a better visualization of the growth of individuals in their different stages of development. The differences between the groups were determined by multivariate analysis (MANOVA). The differences in the redox state, protein oxidation, and western blot analyses were determined by an ANOVA followed by a multiple comparison test of Tukey-Kramer. In all cases, the significance used is mentioned in each figure.

## 3. Results

### 3.1. Morphometric Analysis

To determine if a low-intensity exercise routine for a long period of time (LIERLT) delays the OSO phenotype, the body mass index (BMI) and the body composition were determined in the sedentary (SED) and LIERLT groups during a longitudinal study.

**Figure 1:**
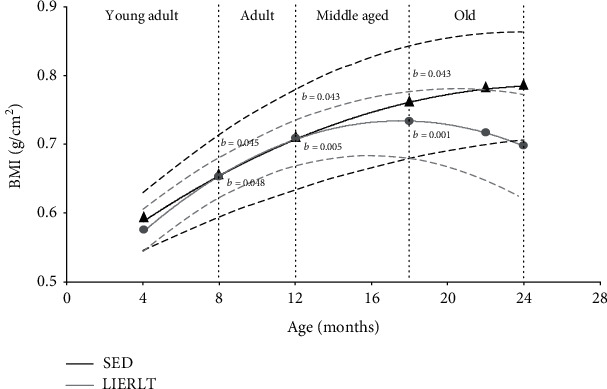
BMI: body mass index. The BMI was calculated as described in Materials and Methods. The dotted lines correspond to the upper and lower limits of the growth curves. Each point in the curve represents the mean ± SD of all independent experiments carried out in each group of animals (*n* = 5). The differences between the experimental groups with respect to the sedentary group (S) are marked with letter *a*, and differences between ages were determined by comparing each age with its predecessor and are denoted as *b*.

To perform a better comparative analysis of the study groups, the data were transformed as population groups and the different stages of rat's development were estimated using polynomial correlations, as shown in Figure 1. The results showed a significant increase of 8% in BMI in both study groups during 4 and 8 months of age (SED, *p* = 0.045; LIERLT, *p* = 0.048). These ages correspond to the rat young adult stage. The following results were obtained between 8 and 12 months of age, corresponding to the rats' adult stage, where an increase of 11% was also observed for both groups (SED, *p* = 0.043; LIERLT, *p* = 0.005). From 12 to 18 months of age is the stage that corresponds to the middle age, and at that stage, an 8% increase was observed in the BMI (SED, *p* = 0.043; LIERLT, *p* = 0.001). In the next stage of development, where the Wistar rats are considered old (from 18 to 24-months of age), no significant changes in the BMI were observed (Figure 1). The BMI results showed no differences between the experimental groups. Subsequently, a body composition analysis was performed using a DXA-scan to evaluate the effects of the LIERLT routine on the body composition of Wistar female rats during aging.

### 3.2. Body Composition (DXA-scan)

**Figure 2:**
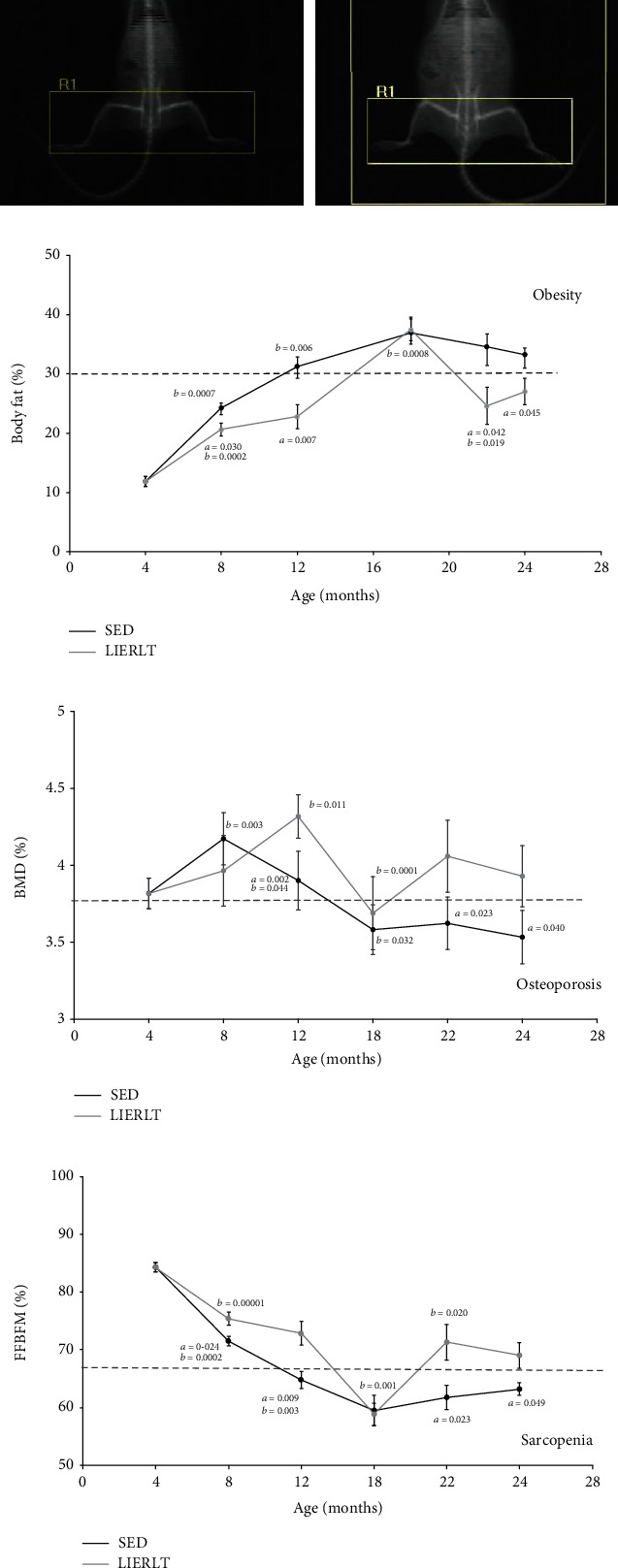
Body composition by dual energy X-ray absorptiometry (DXA-scan). Body composition was determined by DXA-scan; the images show the different areas analyzed: R1 the legs, R2 the arms, and R3 the whole body (a). The determination of the percentage for each of the evaluated parameters was established in Materials and Methods. Percentage of total fat mass (b). Percentage of bone mineral density (BMD) (c). Percentage of lean mass without bone (LMWB) (d). Each point in the curve represents the mean ± SD of all independent experiments carried out in each group of animals (*n* = 5). The differences between the experimental groups with respect to the sedentary group (S) are marked with the letter *a*, and differences between ages were determined by comparing each age with its predecessor and are denoted as *b*.

The body composition evaluation was performed using the dual X-ray absorptiometry (DXA-scan) at the different stages of development described above. The total body composition and the upper and lower extremity composition were evaluated (Figure 2(a)). These values were related to the square of the nasorectal length to determine the skeletal muscle index (SMI). The results in Figure 2(b) show that the animals in the SED group increased their body fat composition in a higher percentage compared to the animals in the LIERLT group, in which a significant increase was observed (14.99% at 8 months, 27.12% at 12 months, 28.84% at 22 months, and 18.67% at 24 months, with of *p* = 0.030, 0.007, 0.042, and 0.045, respectively). Obesity in rats is considered to be established when the animals have a fat percentage greater than 30%, so the rats in the LIERLT group only presented obesity at 18 months, while the SED rats were obese from 12 to 24 months of age. Regarding bone mineral density (BMD), it can be observed in Figure 2(c) that at 8 and 18 months there were no significant changes between the SED and LIERLT groups. Changes in BMD were observed at 12, 22, and 24 months, since LIERLT rats significantly increased their BMD percentage with respect to SED rats in 10.66, 11.87, and 11.04% with a significance of 0.002, 0.023, and 0.040, respectively.

On the other hand, osteoporosis is established when the BMD diminishes 2.5 times the standard deviation with respect to the mean of SED young adult rats (8 months old). Based on this criterion, it can be observed that the SED rats presented osteoporosis since 18 months, whereas the LIERLT rats only presented osteoporosis at 18 months. Next, the lean mass without bone (LMWB) was evaluated, the results in Figure 2(d) show that the rats in the LIERLT group had a higher LMWB than the rats in the SED group, 5.42% at 8 months (*p* = 0.024), 12.47% at 12 months (*p* = 0.009), 15.42% at 22 months (*p* = 0.026), and 9.27% at 24 months (*p* = 0.049). Interestingly, at 18 months of age the SED and LIERLT groups did not show significant differences. Considering the previous results, it was established that sarcopenia in our experimental model was established when the decrease in LMWB was more than 2 times the standard deviation with respect to the mean of the SED young adult rats (8 months). In this way, the SED group rats presented sarcopenia at 12 months of age, while the rats in the LIERLT group delayed the sarcopenia onset to 18 months of age.

### 3.3. Grip Strength

**Figure 3:**
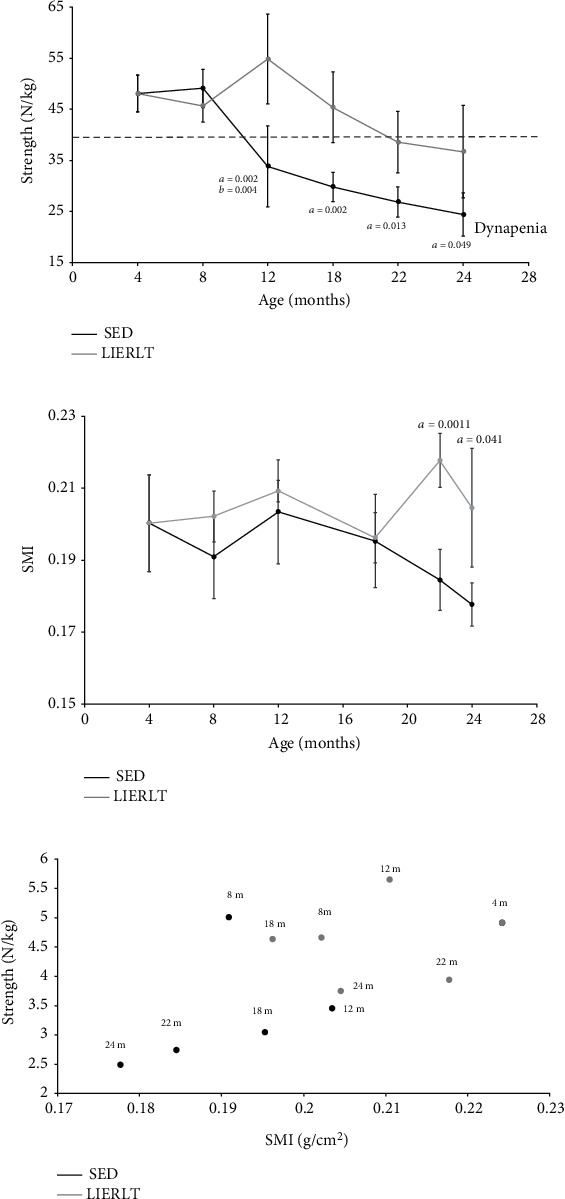
Muscle strength and functionality. We determined grip strength using dynamometry, and the results were normalized with the body mass of each rat: the dotted line is the cut-off point for establishing dynapenia (a). To determine muscle functionality, we determined the composition of LMWB in the arms and legs and related it to nasorectal length, a morphometric parameter known as the SMI muscle mass index (g/cm^2^) (b). Muscular functionality is the ratio of SMI to strength (c). The values represented in the graph are the average of 5 independent determinations. The differences between the experimental groups with respect to the sedentary group (S) are marked with the letter *a*, and differences between ages were determined by comparing each age with its predecessor and are denoted as *b*.

Our results regarding grip strength showed that both experimental groups decreased their force over time (Figure 3(a)). However, from 12 months of age and on, the LIERLT group displayed greater strength than the SED rats; at 12 months, the force increase was 62.12% (*p* = 0.0025), at 18 months; 52.21% (*p* = 0.0023), at 22 months; 43.55% (*p* = 0.013); and at 24 months, 50.59% (*p* = 0.038). The experimental groups did not present significant differences at 8 months of age.

### 3.4. Muscle Functionality

To more precisely determine the muscular composition of the individuals, the skeletal muscle index (SMI) was determined as described in the methodology. The results of this evaluation are shown in Figure 3(b) and reflect that the 22 and 24 months of age rats of the LIERLT group showed a greater amount of muscle than the SED rats (18 and 15.25%, with a significance of *p* = 0.028 and *p* = 0.039, respectively) The groups of 8, 12, and 18 months old did not present significant differences.

Muscle functionality was determined through the relationship between SMI and strength; this functions as a parameter to compare the functionality between the SED and LIERLT groups. The results (Figure 3(c)) showed that SED rats gradually decreased their muscular functionality from 12 months onwards. While LIERLT rats showed a different behavior, at 12 months, LIERLT rats displayed the highest functionality for both groups. However, this functionality declined at 18 months of age. Interestingly, at 22 months of age, the LIERLT rats showed better functionality, although they had less muscle, than the LIERLT rats aged 18 months. Finally, the 24-month-old rats in the LIERLT group of rats had the lowest functionality, which was directly related to aging. An interesting result is that the 24-month-old LIERLT rats showed similar muscular functionality as the 12-month-old SED rats, suggesting that the LIERLT exercise routine delays the onset of sarcopenia.

### 3.5. Biochemical Parameters

The biochemical evaluations related to the metabolic profile of the experimental groups are displayed in Table 1. Interestingly, all the measurements fall within of the normal values established for healthy rats of their respective ages. However, the creatinine levels decreased with age in SED rats in counterpart with the LIERLT groups that did not show significant differences in the evaluated ages. A decrease in serum creatinine has been related to a decrease in skeletal muscle mass, so the results of the decrease in LMWB found in SED rats might be related to this decrease in serum creatinine.

### 3.6. Inflammatory Profile

**Figure 4:**
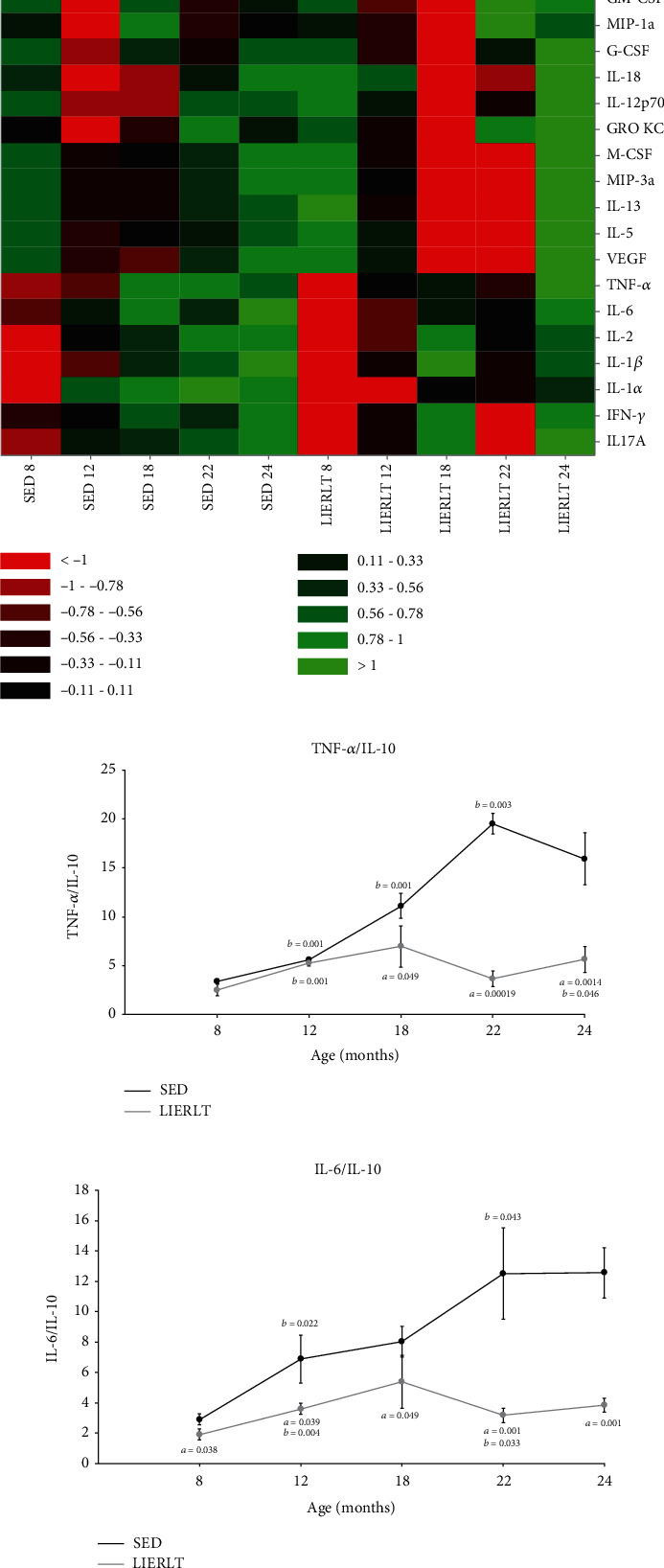
Inflammatory profile. The inflammatory profile was determined using the Bioplex assay. The concentrations of different cytokines: IL-1*α*, IL-1*β*, IL-2, IL-4, IL-5, IL-6, IL-7, IL-10, IL-12p70, IL-13, IL-17A, IL-18, MCP-1, MIP-1a, MIP-3a, M-CSF, IFN-*γ*, G-CSF, GM-CSF, GRO-KC, RANTES, VEGF, and TNF-*α*, were evaluated, ratios in the serum of female Wistar rats at 4, 8, 12, 18, 22, and 24 months of sedentary and exercised age (a). Ratio TNF-*α*/IL-10 (b) and ratio IL-6/IL-10 (c). Values presented are the mean ± SE of independent experiments (*n* = 5). The differences between the experimental groups with respect to the sedentary group are marked with the letter *a*, and differences between ages were determined by comparing each age with its predecessor and are denoted as *b*.

The 23 cytokines described in the methodology were evaluated; the concentrations obtained and normalized were plotted on a heat map in Figure 4(a). The results showed that both groups gradually modified the cytokine concentration throughout life. In particular, there was an increase in the proinflammatory cytokines IL-1*α*, IL-1*β*, IL-6, and TNF-*α* and a decrease in the anti-inflammatory cytokines IL-4 and IL-10. Additionally, it was observed that some cytokines presented an oscillatory behavior in the SED and LIERLT groups throughout life, as in the case of the following cytokines: MCP-1, RANTES, IL-7, GRO KC, M-CSF, MIP-3a, IL-13, IL-5, and VEGF. Since this analysis did not allow us to visualize the effect of LIERLT routine on the inflammatory profile, to discern between the pro- and anti-inflammatory responses, two ratios were made that relate the concentration of proinflammatory cytokines with respect to the anti-inflammatory ones in both the experimental groups. We chose three of the main pro- and anti-inflammatory cytokines that have been related to the inflammatory profile in OSO, TNF-*α*, IL-6, and IL-10 and performed diverse quotients. The ratios obtained were interpreted in the following way: the higher the value, the greater the proinflammatory state. The first ratio evaluated was TNF-*α*/IL-10; the results showed that SED rats gradually increased their inflammatory state until 22 months of age; this state remained constant until 24 months, while LIERLT rats maintained their inflammatory state practically constant throughout life. When comparing the experimental groups, it is observed that the rats of the LIERLT group decreased the proinflammatory profile with respect to the SED rats (Figure 4(b)). In particular, there was a decrease of 38% at 18 months (*p* = 0.049), of 72% at 22 months (*p* = 0.00019), and of 65% at 24 months (*p* = 0.046). The relationship between IL-6 and IL-10 was also evaluated. The results were very similar to those obtained for TNF-*α*/IL-10, a gradual increase in SED rats and constant values of the IL-6/IL-10 ratio. However, when comparing the proinflammatory ratios at the different ages sampled, it was found that the LIERLT exercise routine decreased the inflammatory profile at all ages: 34% at 8 months (*p* = 0.038), 48% at 12 months (*p* = 0.039), 33% at 18 months (*p* = 0.049), 75% at 22 months (0.033), and 70% at 24 months (*p* = 0.001), suggesting that low-intensity exercise throughout life decreases the proinflammatory profile (Figure 4(c)).

### 3.7. GDF-11 Serum Concentration

**Figure 5:**
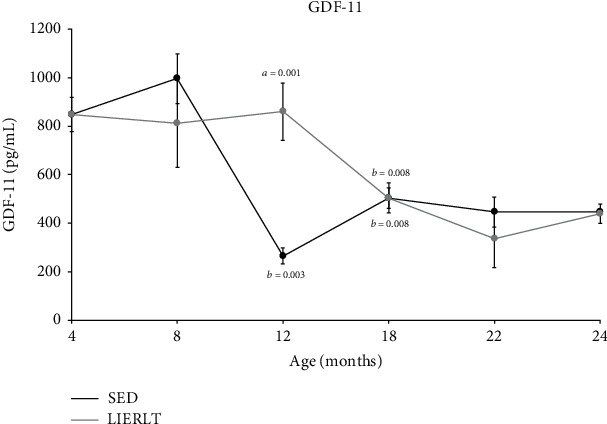
GDF-11 in serum. The concentration of GDF-11 was determined in the serum of female rats of different ages 4, 8, 12, 18, 22, and 24 months of age sedentary and exercised by ELISA as mentioned in Materials and Methods. Values presented are the mean ± SE of independent experiments (*n* = 5). The comparisons were established by a multivariate test and corroborated with a Student *t*-test. The differences between the experimental groups with respect to the sedentary group are marked with the letter *a*, and differences between ages were determined by comparing each age with its predecessor and are denoted as *b*. Significance is specified in the graphs.

GDF-11 concentration was evaluated in animal's serum. The results indicated that in both groups GDF-11 serum concentrations decreased throughout life. However, an interesting difference was observed between both groups; LIERLT rats showed a GDF-11 higher concentration between 12 and 18 months of age compared to SED rats. Therefore, it is possible that this difference in GDF-11 serum concentration might be one of the factors inducing muscle functionality improvement and decreasing body fat in LIERLT rats (Figure 5).

### 3.8. Morphometric and Biochemical Parameters: Population Analysis

To establish a possible relationship between the different morphometric and biochemical parameters and GDF-11 levels, a comparative analysis of the entire population of SED and LIERLT rats from 8 to 24 months of age was performed. The results showed that the LIERLT rats had 7.32% more LMWB (*p* = 0.021), 5.52% more BMC (*p* = 0.014), and 16.10% less fat (*p* = 0.019) than the SED group of rats. With regard to muscle functionality, the LIERLT rats had 33.68% more strength than the SED rats (*p* = 0.0003). With respect to the biochemical parameters related to muscle mass loss, repair, and regeneration (Table 2), it was observed that serum creatinine levels were higher in LIERLT rats (33.33%, *p* = 0.0007) with a GDF-11 content at 14.77% (*p* = 0.031) with respect to SED rats.

### 3.9. Redox State and Protein Oxidative Damage

**Figure 6:**
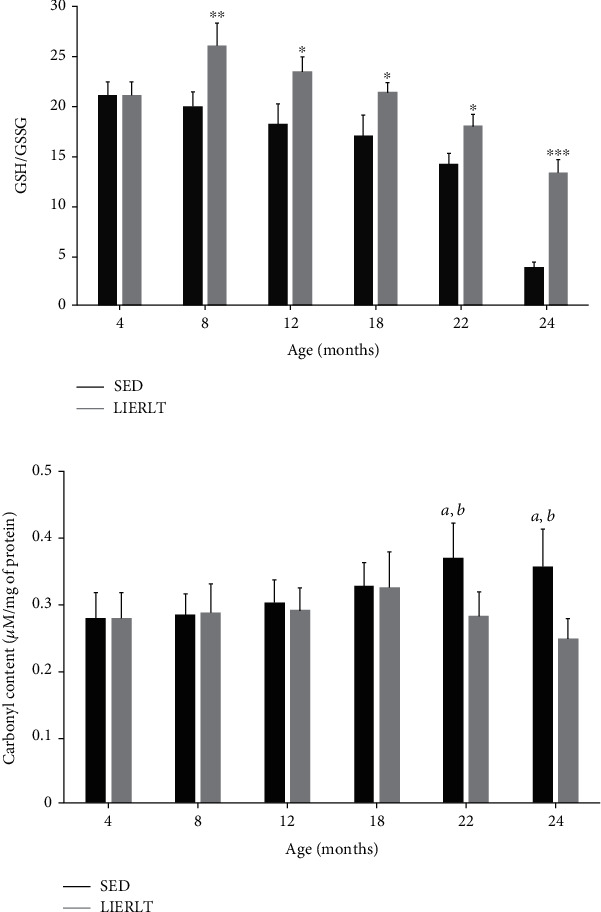
Redox state and oxidative damage. The redox status was determined by quantifying the GSH/GSSG ratio. The concentrations of reduced glutathione (GSH) and oxidized glutathione (GSSG) were determined by HPLC using the gastrocnemius muscle obtained from animals of the two experimental groups at their euthanasia (4, 8, 12, 18, 22, and 24 months), as described in Materials and Methods (a). The GSH/GSSG ratio is the mean ± S.D. (*n* = 5). Oxidative damage was determined by quantifying oxidized proteins. The determination was carried out spectrophotometrically by determining the carbonyls in the proteins as detailed in Materials and Methods (b). Comparisons were made using a Student *t*-test. The significance value is indicated in each group (^∗^*p* = 0.05, ^∗∗^*p* = 0.01, and ^∗∗∗^*p* = 0.001).

To determine the redox state, the GSH/GSSG ratio was obtained, and significant differences were found from 8 months of age in the study groups. The LIERLT rats presented a more reduced redox state, defined by a higher GSH/GSSG ratio, as observed in Figure 6(a), which was greater than that of the SED rats by 30.51% at 8 months (*p* = 0.0012), 28.91% at 12 months (*p* = 0.0065), 25.89% at 18 (*p* = 0.0078), 26.53% at 22 months (*p* = 0.0091), and 250% at 24 months (*p* = 0.00001). These results agree with the protein oxidative damage, since at 22 and 24 months of age, the SED rats presented a higher level of protein carbonylation compared to the LIERLT rats, at 31.10% for the 22-month-old rats (*p* = 0.0001) and 44.11% in the 24-month-old rats (*p* = 0.0001) (Figure 6(b)).

### 3.10. Kaplan-Meier Curve

**Figure 7:**
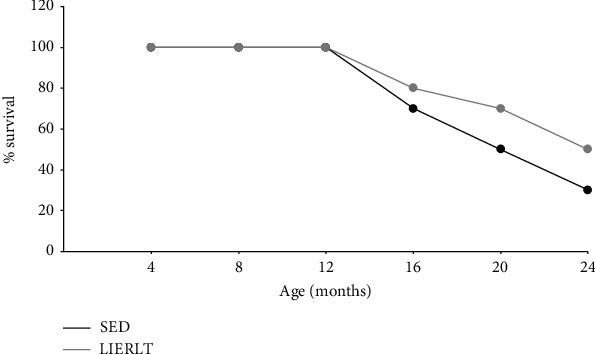
Kaplan-Meier survival curve. To evaluate the effect of exercise routine LIERLT on animal survival, a Kaplan-Meier curve was performed. The total number of evaluated animals was one hundred five.

Finally, to determine the beneficial effect of the low-intensity exercise during 24 months, survival was determined using a Kaplan-Meier curve in the SED and LIERLT groups. The animal survival evaluation showed that reducing body fat and delaying muscle mass functionality loss directly affects the rate survival. Possibly in a mechanism that involves an increase in the GSH/GSSG ratio and a decrease in oxidative damage related to an increase in GDF-11. The results in Figure 7 show that the SED group had only a 30% survival at 24 months of age, while the LIERLT rats showed a 50% survival, which represents an increase in the survival rate of 66.66%.

## 4. Discussion

Numerous studies have shown beneficial effects of performing physical activity during aging, among which are muscle tone improvements, cognitive function recuperation, metabolism activation, inflammatory profile reduction, and a muscle mass loss delay. Hence, exercise has been considered an antiaging intervention [39, 40]. The exercise programs most widely implemented include diverse routines such as high- or moderate-intensity training, aerobic, resistance, and muscle relaxation techniques. These exercise routines have shown their beneficial effects in 3- to 12-month programs, in both humans and rodents [39–43]; however, there are no studies for long term exercise routines. In this study, we demonstrated for the first time the beneficial effects of practicing a low-intensity exercise routine for a long time (LIERLT), which was performed by female Wistar rats during 30 minutes, 5 days a week for 20 months. The LIERLT routine has shown to delay the onset of the osteosarcopenic obesity (OSO) during aging. There are reports in older adults, where performing some type of low-intensity activity for 150 min a week, for long periods of time, increased life expectancy and health, mainly by reducing the risks of falls and injuries that individuals may present when practicing high-intensity exercise routines [44]. The foregoing suggests that adapting the LIERLT routine into practice in middle-aged and elderly humans could have great health benefits.

OSO is a complex syndrome that includes bone functional deterioration (osteopenia/osteoporosis), muscle mass and strength decrease (sarcopenia), and body fat augmentation. These modifications are mainly related to the body changes that occur during aging and possibly due to the effect of other diseases. OSO is associated with a low physical performance, frailty, immobility, falls, fractures, and disability [33, 34, 45]. There are few rodent models to study OSO, and most of them induce obesity in adult female organisms by performing an ovariectomy to observe the effects on muscle and bone during aging [35, 46]. It has been reported that when rats and mice are raised under standard laboratory conditions, with ad libitum access to food and little stimulatory environment for movement, they tend to develop obesity, hypertension, and insulin resistance [28, 29]. Animals in sedentary conditions increase their body fat between 30 and 50%, without developing metabolic alterations, and they do not increase their glucose plasma concentration, total cholesterol, and LDL. These results might suggest a metabolic adaptation to obesity. In humans, a similar effect has been reported in a particular state of obesity or overweight that does not present metabolic alterations. This state is known as metabolically healthy overweight/obesity (MHOW) and has a prevalence of 36% in young adults [47, 48]. A recent study that analyzed a genomic microarray in MHOW individuals showed an overexpression of several pathways related to metabolic processes, which might partially explain the adaptation process of individuals to obesity or overweight.

In counterpart with the metabolic data, sedentary animals have shown high concentrations of proinflammatory cytokines such as IL-6 and TNF-*α* [49]. The above characteristics are consistent with our model of OSO associated with sedentarism and aging, since the rats in the SED group increased their fat content above 30% at 12 months of age, without showing alterations in the biochemical parameters related to metabolism (Table 1). SED rats also increased the expression of proinflammatory cytokines, among which are IL-6 and TNF-*α*.

Based on previous studies carried out in rats and humans [50, 51], and taking into account our results, it is possible to propose some cut-off points with which characterize the OSO model in female Wistar rats used in this study. Along with obesity, osteoporosis, sarcopenia and dinapenia, animals in our OSO model also presented an increase in the inflammatory profile throughout life and an increase in oxidative stress, which is why we consider that this model of OSO resembles many of the clinical characteristics observed in OSO in human.

The animals subjected to the LIERLT routine decreased their body fat content, as well as the inflammatory profile and oxidative stress; they also increased the BMD and delayed the onset of sarcopenia compared to SED rats during aging. One of the main advantages of LIERLT is that it can be performed by all aging organisms, including those with obesity, lack of physical conditioning, and limited mobility. Different authors have recommended that exercise routines in rats over 18 months of age should not exceed 15 m/min, which is equivalent to a low-intensity exercise routine [52–54]. Performing exercise routines with speeds greater than 15 m/min can cause exhaustive exercise in old rats, which increases oxidative stress and damage to the skeletal muscle, heart, liver, lung, and kidney [55, 56].

Age-related fat gain is known to correlate with muscle and bone deterioration during OSO [35, 46]. Moreover, it has been observed that body fat increases in Wistar rats throughout life, which has been related to a decrease in muscle mass, BMC, and life expectancy [57–60]. As mentioned before, one of the main benefits observed in the LIERLT routine was that reducing body fat increased the survival of Wistar female rats by 66%. This body fat reduction might be involved in physiological and molecular mechanisms related to the decrease of the inflammatory state, along with the increase in the antioxidant response and the expression of proteins related to muscle regeneration, such as GDF-11.

The excess of adipose tissue has also been related to an increase in the inflammatory state and oxidative stress. Obese rats have been reported to augment the concentrations of proinflammatory cytokines such as IL-6, IL-1*β*, and TNF-*α*, in addition to increased levels of ROS and malondialadehyde and decreased GSH and GSH/GSSG ratio [61, 62]. Specifically, IL-6 and TNF-*α* high concentrations have been directly related to the process of sarcopenia and frailty in both humans and rats [43, 63–65]. Lower concentrations of proinflammatory cytokines, specifically IL-6 and TNF-*α*, were found in the serum of rats subjected to LIERLT. Furthermore, an increase in the anti-inflammatory cytokines IL-4 and IL-10 was observed. To corroborate that the LIERLT routine reduces the inflammatory profile, we determined the ratio proinflammatory/anti-inflammatory cytokine concentrations using the ratios TNF-*α*/IL-10 and IL-6/IL-10. This strategy allowed us to assess the induction of an inflammatory state and the ability of the organisms to generate a compensatory response, suggesting that one of the physiological mechanisms that are improved with LIERLT is the reduction of the inflammatory state, which is consistent with different reports, in which it has been observed that exercise decreases the expression of inflammatory markers related to obesity, such as NF-*κ*B, IL-6, and TNF-*α*, C-reactive protein, and cyclooxygenase 2 (COX2), in obese and aged Sprague Dawley rats [66, 67]. Diverse studies had been reported a highly proinflammatory environment concurs with greater oxidative damage, mainly by ROS-generating enzymes such as NOX2 and peroxiredoxin 2 (PRDX2) that are increased [68–70], whereas antioxidant enzymes, such as superoxide dismutase and glutathione peroxidase, and GSH plasma concentrations, are diminished. In a model of obesity induced with a hypercaloric diet in Sprague Dawley rats, an increase in proinflammatory markers such as IL-1*β* and TNF-*α* was also observed along with oxidative stress [71]. This same relationship of proinflammatory environment and increased oxidative stress can be observed during aging. A study carried out by Annunziata and collaborators showed a lower activity of catalase, glutathione reductase, and glutathione peroxidase in the gastrocnemius muscle of old Sprague Dawley rats, along with an increase in oxidative stress biomarkers such as malondialdehyde and nitrotyrosine, related to a proinflammatory state determined by the increase in IL-6 expression [72]. Our results indicated that the LIERLT routine reduced the oxidative stress since the animals that exercised presented a higher GSH/GSSG ratio than SED rats, and at the same time, had less oxidative damage to proteins.

Since exercise has been proposed as an antiaging factor, the levels of GDF-11, a protein related to the regeneration and repair of muscle tissue, were evaluated. GDF-11 levels are known to decline throughout life, and this has been related to the decline in physiological, biochemical, and structural functions during aging, mainly in the regeneration of skeletal muscle [25, 73]. Rats in the LIERLT group exhibited lifelong elevations in GDF-11 serum levels compared to the SED group, according to what has been reported in humans and mice [24, 26, 73, 74]. Interestingly, in the study by Elliot et al., the sedentary individuals had a higher percentage of body fat, suggesting a possible relationship between GDF-11 levels and body fat deposits. Here, we observed that, in both study groups, the body fat percentage of increased with age. However, the LIERLT rats always presented a lower fat percentage than the SED, with the exception of 18 months of age, where no significant differences were observed. Interestingly, between 12 and 18 months of age a significant decrease in GDF-11 was found in LIERLT rats, reinforcing the idea that GDF-11 expression could be related to the percentage of body fat. Furthermore, our results showed that between 12 and 18 months of age the survival of the SED rat population decreased. These results suggest a relationship between GDF-11 levels with health, survival, and longevity. This is supported by studies in mice, where it has been observed that GDF-11 elevated levels were indicative of longer lives [75]. Furthermore, it was observed that GDF-11 increases with age in healthy individuals [76]. It is important to emphasize that between 12 and 18 months of age, female Wistar rats present a transition between adulthood and old age, so that in this period a change is observed in the hormonal profile and in the mechanisms of epigenetic regulation [77]. However, more studies are needed to elucidate the role of GDF-11 in health, survival, and longevity.

Another hypothesis to explain LIERLT's beneficial effects might be linked to molecular mechanisms that relate GDF-11 levels to oxidative stress. Recently, it has been reported that exogenous administration of recombinant GDF-11 (rGDF-11) reduced myocardial damage after an ischemia-reperfusion event improving cardiac function, in a Sprague-Dawley rat model; the mechanism proposed implied an increase in the expression of hemoxygenase 1 (HO-1) and a decrease in oxidative damage [78]. Another work reported that rGDF-11 administration for 4 weeks to old mice decreased lipid and protein oxidation by increasing catalase, glutathione peroxidase, and superoxide dismutase antioxidant activities [79], proposing that GDF-11 antiaging function could be related to its antioxidant function. This is consistent with our results that showed a correlation among the higher GDF-11 concentrations evaluated in the LIERLT rat plasma and the less oxidative protein damage and a lower redox stated rats. The decrease in oxidative damage brings with it less muscle tissue deterioration, which implies a greater amount of healthy and functional tissue and an increase in animal's survival. Nevertheless, it is still necessary to carry out more experiments to demonstrate GDF-11 participation within the antioxidant response and to elucidate the molecular mechanisms that are activated with the LIERLT exercise routine, mainly those that respond to redox state modifications, antioxidant response, and GSH metabolism.

In summary, we have reported for the first time the benefits of the LIERLT routine training for 20 months in a model of Wistar female rats. The LIERLT routine favorable effects were observed in each of the life stages of the rats, since there was an increase in muscle mass and strength, as well as BMD and antioxidant response. The molecular protective mechanisms might are related to an increase in the rejuvenation protein GDF-11 and a decrease in body fat and inflammation. An additional contribution is the proposal that the LIERLT exercise routine can be performed in individuals with low physical performance and with overweight or obesity and will still help promoting a better quality of life during old age.

## Figures and Tables

**Table 1:** Biochemical analysis. The content of the biochemical parameters evaluated at 4, 8, 12, 18, 22, and 24 months, when the animals were euthanized.

Age (months)	Glucose (89-163 mg/dL)	Creatinine (0.3-0.6 mg/dL)	GPT (64-222 U/L)	GOT (14-64 U/L)	GGT (8.8-32 U/L)	T-chol (23-97 mg/dL)	HDL-c (32.8 mg/dL)	TG (16-175 mg/dL)
	Sedentary							
4	127.8 ± 2.6	0.8 ± 0.12	158 ± 52.8	31.8 ± 5.07	13 ± 3	59 ± 8.6	18.6 ± 6.8	71.2 ± 26.6
8	114.2 ± 2.7	0.66 ± 0.05	336 ± 51.5	35.6 ± 2.9	10.2 ± 0.5	74.4 ± 14.4	21.2 ± 4	179.6 ± 46.7
12	139 ± 9.17	0.67 ± 0.06	147.67 ± 24.03	20.67 ± 2.08	10.67 ± 1.2	90.67 ± 34.5	27 ± 14	88.67 ± 15.3
18	113.5 ± 9.7	0.75 ± 0.1	142.25 ± 30.3	20.25 ± 7.3	10 ± 0	86.25 ± 19.2	27.25 ± 6.3	84.25 ± 26.3
22	74.33 ± 3.5	0.23 ± 0.06	38.33 ± 6.5	21.67 ± 8.08	15.67 ± 8.2	71.67 ± 6.8	23.33 ± 2.5	141.33 ± 23.7
24	101.5 ± 46	0.2 ± 0^∗∗∗^	82 ± 48.5^∗∗^	18 ± 11.3	10 ± 0	74.5 ± 29	32 ± 15.7	80 ± 38.2
	Exercise LIERLT							
8	131 ± 14.09	0.66 ± 0.06	283.6 ± 75.5	36.4 ± 12.7	10.8 ± 1.8	75.2 ± 8	26.4 ± 2.5	99.6 ± 26.05
12	109.5 ± 9.5	0.75 ± 0.06	163.75 ± 76.4	55.75 ± 38.2	10 ± 0	100.25 ± 8.4	29 ± 5.5	126.75 ± 57.3
18	107 ± 13.4	0.8 ± 0.08	149.75 ± 24.4	27.5 ± 11.39	12 ± 1.4	84.5 ± 51.1	23.5 ± 12.5	190 ± 212
22	109.25 ± 9.3	0.78 ± 0.095	164.75 ± 36	20.75 ± 9.6	11 ± 1	67 ± 13.6	20.75 ± 4.1	211.75 ± 128.5
24	92.8 ± 24.2	0.62 ± 0.08^∗∗∗^	183.2 ± 28.2	26.6 ± 14.6	11.4 ± 1.2	107.4 ± 27	32 ± 9	117.6 ± 20.67

The experimental results are compared to the ranges reported by the clinical laboratory parameters for Wistar rats, named test in the table. To compare the groups, the ANOVA test was used followed by a Tuckey-Kramer post hoc test. The significance value is indicated in each group (^∗^*p* = 0.05, ^∗∗^*p* = 0.01, and ^∗∗∗^*p* = 0.001).

**Table 2:** Comparison of body composition and GDF-11. Body composition and GDF-11 concentration were compared in exercised and sedentary animals from 8-24 months of age.

Variable	Sedentary 8-24 months	Exercise LIERLT 8-24 months	Probability
BMI	0.727 ± 0.098	0.688 ± 0.085	0.165
Strength	33.84 ± 10.03	45.24 ± 9.41	*0.0003*
Creatinine	0.54 ± 0.22	0.72 ± 0.08	*0.0007*
GDF-11	543.06 ± 171.96	623.30 ± 142.56	*0.031*
%FFBFM	65.26 ± 6.31	70.04 ± 7.07	*0.021*
%Fat	30.92 ± 6.53	25.94 ± 7.21	*0.019*
%BMC	3.80 ± 0.28	4.01 ± 0.28	*0.014*

The values represented the mean ± SE of independent experiments (*n* = 25). Comparisons were made using a Student *t*-test. The differences between the experimental groups with respect to the sedentary group are marked, and the significance is specified.

## Data Availability

All the data supporting the results of our study are shown in the figures and tables within the manuscript.
